# Processing of High-Performance Polymeric Materials: Modeling and Characterization

**DOI:** 10.3390/polym17060783

**Published:** 2025-03-15

**Authors:** Christoph Burgstaller, Gernot Zitzenbacher

**Affiliations:** 1 Transfercenter für Kunststofftechnik GmbH, Franz-Fritsch-Str. 11, 4600 Wels, Austria; 2 School of Engineering, University of Applied Sciences Upper Austria, Stelzhamerstraße 23, 4600 Wels, Austria

## 1. Introduction

Polymers are an integral part of our world. While polymers used en masse, like the ones used for packaging, are under pressure due to their potential for generating large waste volumes if not managed properly, high-performance polymers play an important role in various applications. Mechanical and thermal properties realized in lightweight fiber-reinforced materials for mobility, high-pressure loaded parts for gas storage for the energy transition, or high tenacity fibers are often unique to polymers and can only be realized with polymer-based materials and composites.

## 2. Overview of the Published Articles

With this Special Issue, we wanted to show the variability of different applications in the field. While short fiber reinforcement for high-load-bearing applications is a rather obvious and well-known application [1,2,3], improvements can still be achieved via proper processing (Contribution 1). Also, PPS as a material for hydrogen compressor pistons (Contribution 2) and thermoplastic elastomer composites for bipolar plates (Contribution 3) are energy-specific applications, helping in moving the energy transition forward. Simulation is a potent tool for understanding composite behavior in loading and processing [4,5,6,7], and can often be used to predict a material’s performance without the need to physically investigate every iteration, therefore cutting down on the number of investigated samples and improving efficiency and development speed. Other simulative approaches, may these be for processing, e.g., heating thermoplastic composites before processing (Contribution 4) or the improved modeling of thermal conductivity (Contribution 5), help us to understand processes better to improve parameters like processing speeds and reducing the number of failed parts. Also, determining the mechanical behavior of polymers while tensile loading (Contribution 6) helps to improve our understanding of polymers, which can further translate into novel applications for specific polymers.

When evaluating the mechanical properties of a composite, several influences need to be considered. One of the approaches is to utilize Weibull statistics, as is performed for evaluating fiber tensile properties in composites, as the fibers are the major load-bearing factor and therefore influence failure behavior most [8,9]. Here, further insight is contributed by investigating the applicability of different distributions (Contribution 7). 

Not only fibers are considered for composite reinforcement. In addition, more filler-like alternatives can improve the properties of an unreinforced thermoplastic [10,11,12,13]. One contribution investigated the utilization of micro silica particles and its influence on mechanical properties (Contribution 8), while another reported on modifying PI films with metal oxide particles (Contribution 9).

Alternatives to typical reinforcements like glass and carbon fibers can not only be generated from inorganic materials, but also from organic matter. Several works report on the challenges and the effects of cellulose nanocrystals in polymers [14,15,16,17]. One contribution investigates cellulose nanocrystals from cotton in PVOH films improving mechanical and barrier properties (Contribution 10).

Polymers are also found in biomedical applications. Usually, these are different from the polymers used for structural applications, but enable high potential in functionality [18,19]. One contribution investigated a simplified preparation of hydrogels from silk fibroin (Contribution 11). 

Another field of application for polymers is adhesives, where a variety of different polymers are utilized in different applications. One very widespread type of adhesives is pressure-sensitive adhesives [20,21,22]. One contribution looks further into improving the adhesion in polyurethane and acrylic-based systems (Contribution 12). 

A variety of high-performance polymers are used in biomedical applications, for scaffolds, different tissues, and also drug delivery systems [18,23,24,25] One contribution looks into the melt spinning of polycaprolactone scaffolds (Contribution 13), while another reports on the surface modification of polyetheretherketone for dentistry implants (Contribution 14). 

For any polymer, structure formation is of great importance, as it dictates the final properties and the processability. One contribution looks into this aspect for high-density polyethylene powder (Contribution 15). In 3D printing, polymers are a prominent material choice [26,27,28,29]. Here, one contribution adds to the understanding of the influence of processing on the mechanical strength of 3D printed materials (Contribution 16).

## 3. Conclusions and Outlook

Therefore, it may be concluded that polymers play a vital role in different applications in our everyday lives. Research progressing further in these various topics is also shown in this Special Issue in energy materials, composites, fibers, simulation, and hydrogels. As guest editors, we are confident that this Special Issue made significant contributions in progressing the knowledge and understanding of modern high-performance polymers, and that future research can build on these contributions to deepen the knowledge on high-performance polymers.
